# A Case Report of Acute Flaccid Paralysis Caused by Enterovirus D68 Infection: The Beginning of a Polio-Like Epidemic?

**DOI:** 10.7759/cureus.15625

**Published:** 2021-06-13

**Authors:** Syeda Nafisa, Pulak Paul, Milind Sovani

**Affiliations:** 1 Respiratory Medicine, Nottingham University Hospitals, Nottingham, GBR; 2 Intensive Care Medicine, Sherwood Forest Hospitals NHS Foundation Trust, Mansfield, GBR

**Keywords:** acute flaccid paralysis, respiratory weaning, respiratory failure, broncho-alveolar lavage, enterovirus d68

## Abstract

Enterovirus D68 (EV-D68) is a non-polio enterovirus that occasionally causes respiratory illnesses. EV-D68 infections have occurred over the last couple of years and have a high prevalence worldwide. This virus has recently been linked to acute flaccid paralysis and particularly affects children. We report the case of a young adult who presented with acute neurological manifestations along with respiratory involvement. EV-D68 was detected in the patient’s broncho-alveolar lavage and was followed by a prolonged recovery period. Clinicians should consider EV-D68 infection in the differential diagnosis of acute flaccid paralysis (AFP) and respiratory failure.

## Introduction

Enterovirus D68 (EV-D68) is a non-polio enterovirus that occasionally causes respiratory illnesses. EV-D68 infections have been frequently reported worldwide. This virus has recently been linked to acute flaccid paralysis (AFP), particularly in children [1].

## Case presentation

A 22-year-old previously healthy male experienced acute onset of productive cough and fever (up to 38°C). He also had headache, visual blurring and facial weakness. After two days, his Glasgow Coma Scale (GCS) was reduced to 10 (from 15) and he developed global flaccid paralysis with evidence of bulbar palsy. He was initially treated for presumed encephalitis with intravenous amoxicillin, ceftriaxone and aciclovir therapy. However, five days after admission, his condition rapidly deteriorated and became unresponsive. An arterial blood gas analysis revealed type 2 respiratory failure necessitating intubation and ventilation. Blood tests revealed a white blood cell count of 9.1 x 10^9^/L, C-reactive protein level of 36 mg/L and normal hepatic and renal functions (Table 1). The remaining tests including antinuclear antibody (ANA), anti-dsDNA, anti-neutrophil cytoplasmic autoantibody (ANCA) and other autoimmune screening tests, were negative at this point in time. Serological tests for respiratory viruses were negative for herpes simplex virus, varicella-zoster virus, enterovirus, adenovirus, influenza A and B viruses, parainfluenza virus and respiratory syncytial virus (Table 1).

**Table 1:** Investigation results 1 Antinuclear Antibody 2 Anti-double stranded DNA 3 Anti-Neutrophilic Cytoplasmic Autoantibody

Cerebrospinal fluid analysis			
	Protein	826 mg/L (15–45)	
Cell count	White cells	612/uL (5)	
	lymphocytes	90%	
	Glucose	4.0 mmol/L	
Blood Tests			
Haematology	Haemoglobulin		120 g/L
	White blood cell count		9.1 x 10^9^/L
	Platelet		156 x 10^9^/L
Biochemistry	C-reactive protein levels		36 mg/L (>5 mg/L)
	Urea		5.9 mmol/L
	Creatinine		57 mmol/L
	Sodium		135 mmol/L
	Potassium		4.1 mmol/L
	Alanine aminotransferase test		30 U/L
	Bilirubin		12 Umol/L
	Alkaline Phosphate		100 U/L
Viral PCR	Herpes simplex virus		Negative
	Enterovirus		
	Parainfluenza		
	Adenovirus		
	Influenza A and B viruses		
	Parainfluenza virus		
	Respiratory syncytial virus		
Immunology	ANA^1^		Not detectable
	Anti-dsDNA^2^		
	ANCA^3^		
	Paraneoplastic antibodies		
	Acetylcholine receptor		
	Antiganglioside antibodies		
Microbiology	Blood cultures		No growth
	Hepatitis B and C viruses		Negative
	Epstein-Barr virus		
	Cytomegalovirus		
	HIV		
	Borrelia burgdorferi		

**Figure 1:**
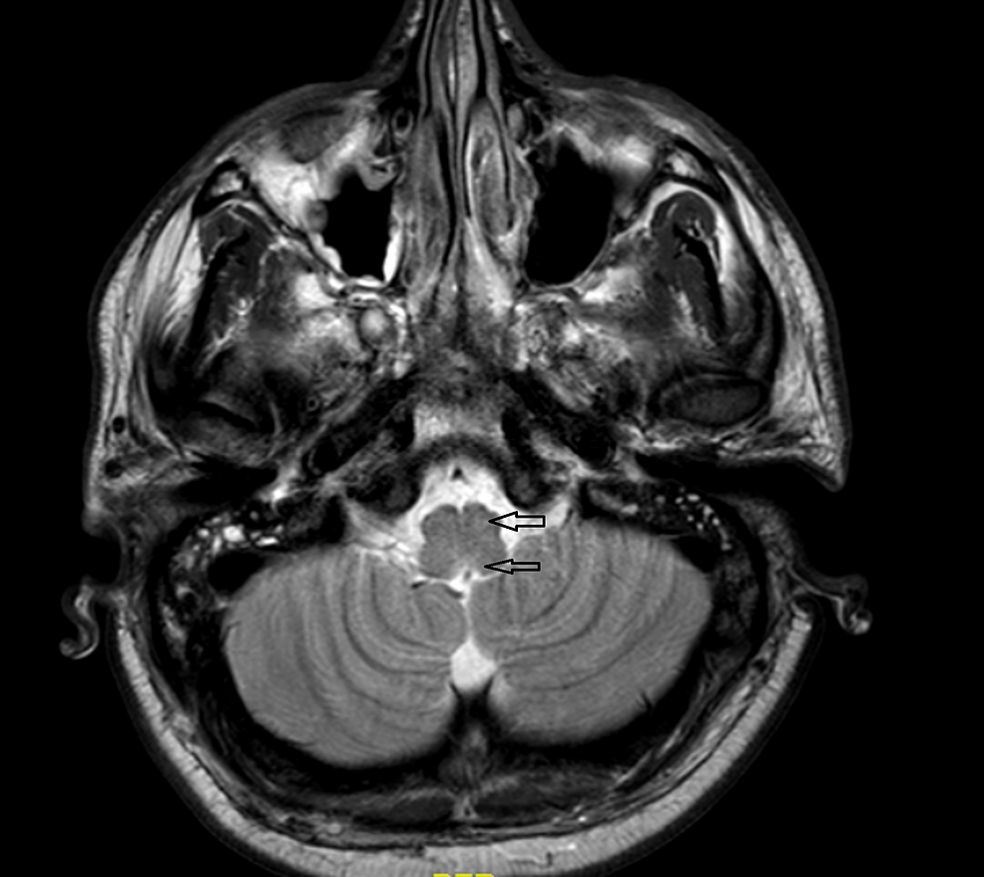
CT scan of cross-sectional view of brainstem showed T2 signal hyperintensity within dorsal brainstem and left cerebral peduncle.

**Figure 2:**
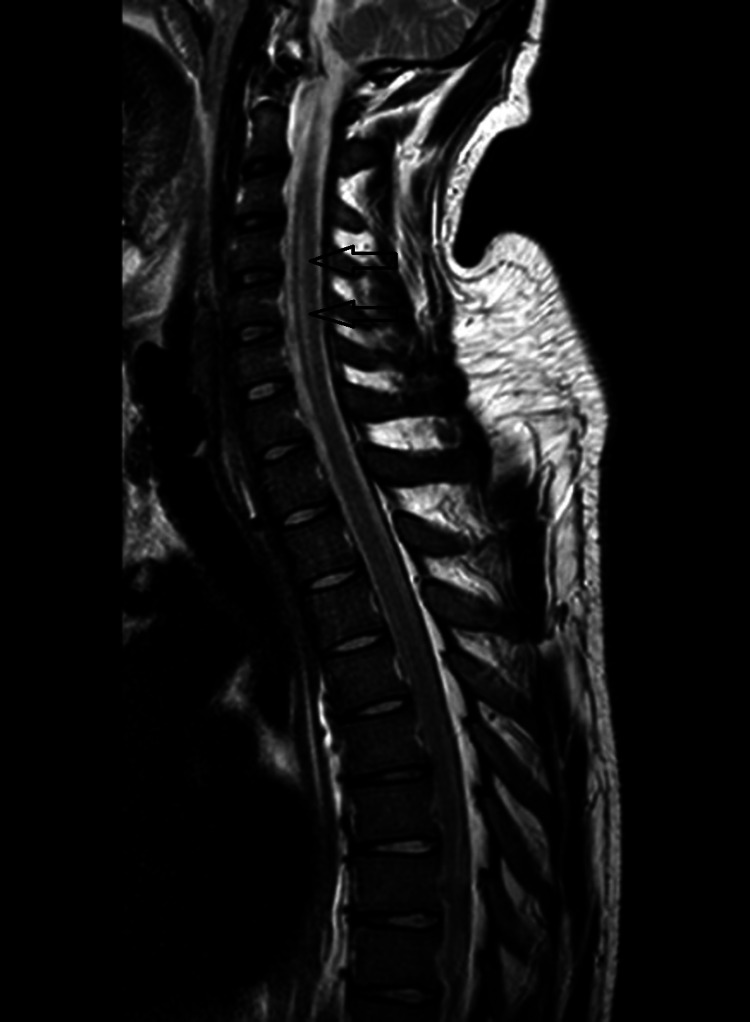
CT scan of sagittal view of cervical and thoracic spine showed T2 signal hyperintensity within anterior horn.

Cerebrospinal fluid (CSF) analysis revealed high protein levels (826 mg/L) and pleocytosis. Magnetic resonance imaging of the brain revealed T2 signal hyper intensity within the dorsal brainstem, left cerebral peduncle and anterior horn (Figures 1, 2).

The patient was transferred to a tertiary neurology centre and was treated with intravenous immunoglobulin and then with 1,000 mg/day of methylprednisolone for three days for presumed Miller Fisher syndrome; however, he did not respond to the treatment. EV-D68 was not detected in the CSF sample, pharyngeal swab, sputum sample or stool samples. However, bronchoalveolar lavage analysis later confirmed the presence of EV-D68. The enterovirus 5′9-UTR-PCR, VP1 genotyping and other standard genotyping could not detect EV-D68 in bronchoalveolar lavage analysis. However, this virus was identified by enterovirus-specific real-time polymerase chain reaction (RT-PCR).

The patient underwent a percutaneous tracheostomy and had a prolonged stay in the Intensive care unit with respiratory weaning. Multiple decannulation attempts were unsuccessful at six months due to undiagnosed diaphragmatic palsy (DP) and sub-optimally managed bulbar weakness. The diagnosis of DP later was confirmed by chest X-ray with elevated diaphragms. A hyoscine patch, atropine drops and tiotropium inhaler were used to control salivary secretion, and the patient was kept ‘nil by mouth’ to prevent aspiration.

Eight months after admission, the patient had an uncuffed tracheostomy but remained on overnight non-invasive ventilation. He has regained limited function of his extremities but requires assistance with all care.

## Discussion

This patient was a young adult and had developed a respiratory tract infection followed by acute flaccid paralysis and respiratory failure caused by EV-D68, with an outcome similar to that of poliomyelitis.

Enterovirus is a single-stranded RNA virus and part of the Picornaviridae family. EV-D68 is slightly different and shares features of both rhinoviruses and enteroviruses. It mostly affects children and usually causes a wide range of respiratory disorders, such as upper respiratory tract infection, severe pneumonia, or even respiratory failure [2, 3]. AFP is the most common neurological presentation in both children and adults [4]. Our patient had bulbar palsy and signal attenuation on MRI, indicating the involvement of the brainstem and the coexistence of myelitis.

In California the incidence of acute flaccid myelitis (that causes AFP) increased from 0.028 to 0.16 per 100,000 people each year and EV-D68 was frequently detected in upper respiratory tract specimen [5]. There has been a report of poliomyelitis-like neurological outbreak in a total of 48 children caused by EV-D68 in North America and Canada in 2014. Neurological weakness, flaccid limb paralysis, bulbar involvement and cranial nerve involvement were the main clinical features. CSF pleocytosis was observed in 10 children, while MRI changes were noted in 10 children [6]. EV-D68 was detected in respiratory specimen samples of five children [3]. Three cases of AFP caused by EV-D68 in children were detected in Europe [4,5]. This virus was isolated from respiratory samples in all three cases. Interestingly, in our patient, EV-D68 was not detected in the pharyngeal swab, sputum or stool sample. Broncho-alveolar lavage analysis confirmed EV-D68. Although CSF analysis showed high protein levels and pleocytosis, the virus was not detected in the CSF. Poliovirus and enterovirus type 71 are neurotrophic viruses, often not isolated from CSF [7]. These viruses are usually isolated from upper respiratory tract or gastrointestinal tract specimens [6]. It is therefore not surprising that EV-D68 could not be detected in the CSF in our patient.

The EV-D68-specific RT-PCR assay used in our case is more sensitive than the EV-D68 RT-PCR assay as per the Centers for Disease Control and Prevention. Moreover, it is highly specific for EV-D68 as it does not amplify other enteroviruses [8]. Hence, EV-D68-specific RT-PCR should be performed in patients with AFP of unknown origin to ensure accurate diagnosis and treatment.

**Figure 3:**
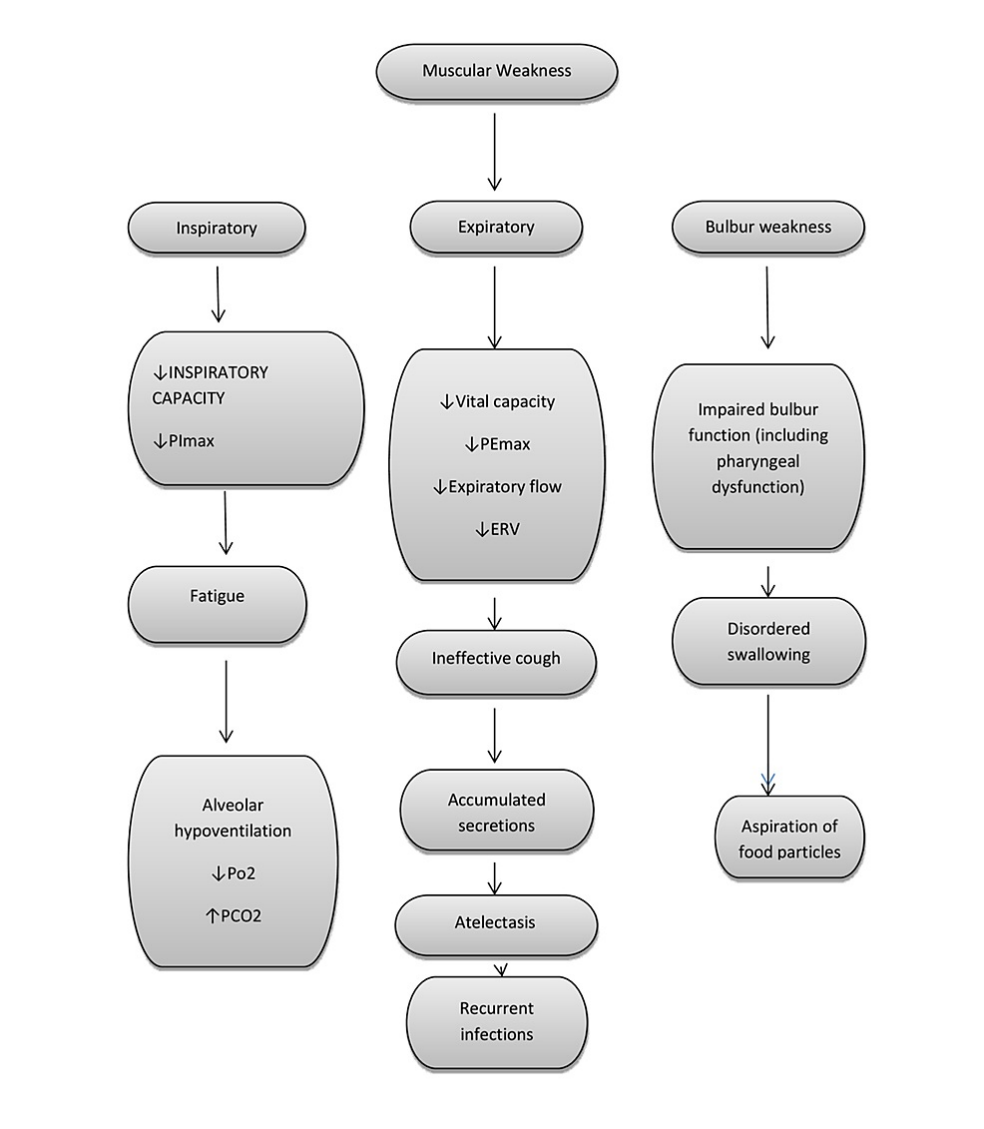
Neuro-respiratory weakness.

The muscles of respiration include inspiratory muscles (mainly diaphragm) that mostly contribute to ventilation, expiratory muscles that are responsible for forced expiration and effective cough and bulbar muscles that guard the airways. The inspiratory capacity and maximum inspiratory mouth pressure (PI max) are reduced in patients with inspiratory muscle weakness. Such patients suffer from a progressive decline in vital capacity (VC), the increase in work of breathing and an inability to take deep breaths which cause a rapid-shallow breathing pattern, leading to alveolar hypoventilation, hypoxia and hypercapnia. Similarly, expiratory muscles weakness associated with inadequate lung inflation reduces vital capacity, maximum expiratory mouth pressure (PE max), expiratory flow and expiratory reserve volume. This prevents effective coughing and airway clearance, changes airway resistance and increases the risk of developing atelectasis and pneumonia [9,10]. Impaired bulbar function (facial, oropharyngeal, laryngeal and pharyngeal dysfunction) can cause abnormal swallowing with the possibility of an increased risk of aspiration (Figure 3). This patient underwent a percutaneous tracheostomy and had a prolonged stay in the intensive care unit with respiratory weaning. Multiple decannulation attempts were unsuccessful due to undiagnosed diaphragmatic palsy and sub-optimally managed bulbar weakness. A hyoscine patch, atropine drops and tiotropium inhaler were used to control salivary secretion, and the patient was kept ‘nil by mouth’ to prevent aspiration.

The United Kingdom AFP Task Force recognized 40 AFP cases in 2018, the larger part influencing children [11]. An enormous number of these patients required critical care admissions, and a noteworthy extent had drawn out neurological deficiency [11]. Although epidemiological studies have unequivocally recommended the relationship among AFP and EV-D68, the natural history of this disease remains elusive, so it is unknown whether certain patients with EV-D68 are more vulnerable to developing AFP [5]. Should the prevalence of EV-D68 rise in the future, the incidence of AFP can rise, with possible significant consequences for critical care and respiratory support for patients affected. Further research is required to clarify the pathophysiology, progression and management strategy for this disease.

## Conclusions

Enterovirus D68 is associated with polio-like acute flaccid paralysis and respiratory failure in adults. We suggest that not only paediatrician but also adult clinicians should think of this EV D68 as a cause of acute flaccid paralysis and respiratory failure and consider sending respiratory samples for specific RT-PCR for EV D68 and bronchoalveolar lavage may be needed in some cases. Assessment of inspiratory, expiratory and bulbar function is necessary before attempting weaning ventilation for getting the optimal outcome.
